# Group-level differences in social network structure remain repeatable after accounting for environmental drivers

**DOI:** 10.1098/rsos.230340

**Published:** 2023-07-19

**Authors:** Mina Ogino, Adriana A. Maldonado-Chaparro, Lucy M. Aplin, Damien R. Farine

**Affiliations:** ^1^ Department of Biology, University of Konstanz, Konstanz 78464, Germany; ^2^ Centre for the Advanced Study of Collective Behaviour, University of Konstanz, Konstanz 78464, Germany; ^3^ Department of Collective Behavior, Max Planck Institute of Animal Behavior, Konstanz 78467, Germany; ^4^ Department of Evolutionary Biology and Environmental Studies, University of Zurich, Zurich 8006, Switzerland; ^5^ Department of Biology, Faculty of Natural Sciences, Universidad del Rosario, Bogota, Cra 26 # 63B – 48, Colombia; ^6^ Cognitive and Cultural Ecology Research Group, Max Planck Institute of Animal Behavior, Radolfzell 78315, Germany

**Keywords:** group-level behaviour, social network, environment, partitioning, repeatability, zebra finch

## Abstract

Individuals show consistent between-individual behavioural variation when they interact with conspecifics or heterospecifics. Such patterns might underlie emergent group-specific behavioural patterns and between-group behavioural differences. However, little is known about (i) how social and non-social drivers (external drivers) shape group-level social structures and (ii) whether animal groups show consistent between-group differences in social structure after accounting for external drivers. We used automated tracking to quantify daily social interactions and association networks in 12 colonies of zebra finches (*Taeniopygia guttata*). We quantified the effects of five external drivers (group size, group composition, ecological factors, physical environments and methodological differences) on daily interaction and association networks and tested whether colonies expressed consistent differences in day-to-day network structure after controlling for these drivers. Overall, we found that external drivers contribute significantly to network structure. However, even after accounting for the contribution of external drivers, there remained significant support for consistent between-group differences in both interaction (repeatability *R*: up to 0.493) and association (repeatability *R*: up to 0.736) network structures. Our study demonstrates how group-level differences in social behaviour can be partitioned into different drivers of variation, with consistent contributions from both social and non-social factors.

## 1. Introduction

Individuals living in social groups often interact with other individuals in consistent ways across time [1], driven by individual differences in sociality [2,3] and non-social factors [4], leading to them having consistent social associates [5] and repeatable positions in their social environment [3]. This concept has further been extended to suggest that social groups can exhibit consistent differences in behaviour (i.e. group cultures, e.g. [3,6]) that can be captured as distinct structural network structures. However, it remains poorly understood what (and in which direction) factors drive these group-specific behavioural patterns, including the relative contribution of social versus non-social behaviours, as well as methodological confounds [7]. Addressing the question of whether groups can exhibit consistent differences in social behaviour (and its corresponding emergent social network structure) independently of external drivers is critical for understanding the emergence of local adaptation [8], animal cultures [9] and social selection [10].

Despite recent technological and statistical developments, determining whether and how two social networks differ remains challenging [11]. A critical open question is whether groups are ever actually expected to have the same network structure under the same conditions (e.g. group size, density and environmental conditions). We know that the social structure of the groups or populations that animals live in are non-random [12], meaning that we cannot assume that two groups or populations will differ randomly. Therefore, to conclude that two groups have consistent differences in social network structure implicitly assumes the null hypothesis ‘all groups experiencing the same conditions have the same social network structures'. The key outstanding question is whether this null hypothesis is plausible, and not invariably be rejected [12,13].

Testing for between-group differences in social network structure requires three steps. First, we need to identify external sources of variations in group-level social network structure. Second, we can quantify how much of the variation in overall network structures (across groups and across time) each source explains. Third, only then can we test whether there remains any evidence for consistent differences in social structure among groups and reject the null hypothesis that groups experiencing the same environment and conditions do not differ in social network structure. While this process is similar to studying between-individual differences, we note that evidence for individual-level differences (e.g. repeatable social network positions within a network, e.g. [3]) does not necessarily mean that the structure of the overall network is consistent (and, conversely, consistent network structure does not necessarily mean that individuals are repeatable in their network positions).

The challenge for studies of between-group differences in social networks is that consistent differences among groups could arise from both social and non-social (e.g. methodological) effects [7]. Unlike studies of between-individual differences in network position or studies investigating whether the specific relationships within a network are consistent over time (e.g. [5]), which are generally conducted on animals often found in the same space, studies of between-group difference are predominately conducted on spatially or temporally separated groups, thereby exacerbating the contribution of external drivers. Here we briefly discuss five broad categories of external drivers that could shape animal social network structure: group size and composition [10], ecological (e.g. temperature, rainfall and availability of cover [14,15]) and physical (e.g. available space or habitat complexity [4]) conditions and differences in methodological approaches (especially insufficient sampling [16]).

Group size inherently produces differences in social network metrics by determining the range of possible values that metrics can take [17]. For example, binary degree (the number of connections an individual has with others) and mean binary degree (how gregarious group members are) cannot be greater than *N* − 1 (where *N* is the number of individuals in the study). Thus, depending on the research question, it might not be meaningful to directly compare some network metrics (e.g. binary degree) between groups of different sizes.

Another source of potential variation is group composition. Previous studies have suggested that how individuals behave and interact with each other can be explained or modulated by traits or experience of individuals themselves (personality [2]; sex [1,3,18]; prior experience [19]; state [20]). The distribution of traits or experience among group members can then shape its properties [10]. For example, groups of *Drosophila melanogaster* with prior social experience formed more connected social networks than groups composed of previously socially isolated flies [19]. Similarly, groups with more aggressive individuals had more clustered social networks [19]. Thus, variation between individuals, and subsequent group phenotypic composition, can produce differences in network structure among groups even when other conditions are identical.

Ecological environmental conditions can also produce differences in group-level social network structures. For example, at individual level, Trinidadian guppies (*Poecilia reticulata*) develop more stable and differentiated social connections under greater perceived predation risk [21] and smaller shoals in turbid water [22], eastern grey kangaroos (*Macropus giganteus*) connect more exclusively with others under higher rainfall [20], and the amount of rainfall affects social interactions in superb starlings (*Lamprotornis superbus*) via stress-mediated mechanisms [23]. At a group level, communities of Masai giraffes (*Giraffa camelopardalis tippelskirchi*) that live closer to humans form more exclusive social relationships with weaker connections [24]. However, while many studies highlight the role of ecological effects on the behaviour of individuals within groups that may lead to specific group-level structures (such as to fission or fusion, [25]), the links between ecological conditions and social network structure remain largely under-studied.

Differences in habitat structure (e.g. connected versus fragmented) or size (e.g. home-range or enclosure size) can also impact social network structure by determining the rate at which individuals are likely to encounter other individuals [26,27]. For example, sleepy lizard (*Tiliqua rugosa*) populations living in artificially more complex environments had higher network density and more stable social connections (i.e. more re-encounters among the same sets of individuals) than populations living in less-complex (i.e. more open) habitats [4]. Since comparisons are often made between populations living in different physical spaces, this easily could account for apparent differences in group-level social networks [27,28].

Finally, methodological differences (e.g. sampling effort) or observation processes (e.g. misidentification of individuals, observer bias) can affect measurement of network metrics, and subsequently the estimation of group-level structure [16]. Observations can be biased towards more easily identified individuals (e.g. individuals with more markings or a brightly coloured sex), which may be especially the case when researchers observe animals under field conditions. Even with systems that automate the identification or tracking of individuals, mistakes or biases can rarely be completely avoided. For example, passive integrated transponder (PIT)-tags may not be detected on radio frequency identification (RFID) antennae under certain body postures (D.R.F., personal observation); markers used to track birds can be covered by feathers [29]; and GPS signal accuracy can be impacted by tree cover, affecting the estimates of social associations [27,30]. Finally, data could be collected at different time scales (e.g. overlapping reproductive seasons versus not, or for different period lengths resulting in different amounts of data contributing to each network). Thus, even when the same observational method is applied across individuals or groups, variation in identification rates and biases are often inevitable. These differences in how observations are made should be always expected to play some role in shaping the structure of social networks across most studies [7].

In this study, we investigate the baseline expectations for group-level differences in behaviour by quantifying how much and why the emergent group-level social network structure—using seven commonly used network metrics—varies across groups. Our aim is not to ask whether individuals have the same social relationships day-in day-out, but rather whether the emergent structure of a given group can differ consistently to that of others (meaning that a substantial proportion of the variance in a given network metric is explained by the identity of each colony). Achieving this requires a system in which: (i) multiple groups can be observed (between-group replication), (ii) groups can be observed repeatedly (within-group replication), and (iii) robust observation methods allow methodological differences to be quantified and controlled for. We achieve this by employing high-resolution tracking to measure daily social networks across 12 colonies of captive zebra finches, each studied in two different seasons. We then estimate the contributions of colony size and composition, environmental (weather and physical) conditions and methodological differences across days and among colonies, by partitioning variation in network structure across these external drivers. While these drivers explain a high proportion of the variance in social network metrics observed among colonies, we still find consistent effects of group (colony) identity on network structure. Our study thereby generates a better understanding of how between-group-level differences in social behaviour arise and demonstrates that even after accounting for external drivers, we can still detect consistent group-level differences in behaviour between groups.

## 2. Methods

### 2.1. Study system

**Figure 1.**
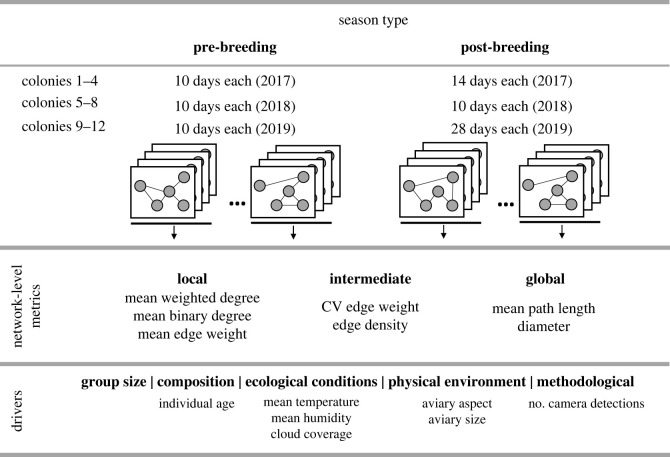
Summary of data structure. This study includes 3 years (2017–2019) and we had four colonies for year. Each colony for each year has 10 days from pre-breeding season. For post-breeding seasons, we used 14, 10 and 28 days of data in 2017, 2018 and 2019, respectively. We had seven colony-level social network metrics (local, intermediate and global-scales), and these metrics were determined by daily social networks for each colony for each network type. For each day, we also determined external drivers which each colony experienced. Both interaction and association networks had the same data structure. We replicated our analysis by keeping pre- and post-breeding seasons separate from each other.

Our 12 captive zebra finch colonies were studied in sets of four colonies per year over 3 consecutive years (2017–2019; figure 1*a*). Colonies were housed in outdoor aviaries at the Max Planck Institute of Animal Behaviour, Radolfzell, Germany with water and food provided ad libitum. Animal care (e.g. cleaning aviaries) was equal for all colonies, and data were collected continuously during these periods. Each colony started with 28 individuals (equal sex ratio), but colony sizes varied due to deaths or birds being taken out for veterinary treatment (26–28 in pre-breeding and 22–28 in post-breeding seasons).

Birds were kept as part of a long-term experiment [5] that simulated three seasonal contexts: pre-breeding (no nesting opportunities), breeding (nest-boxes and nesting materials were available) and post-breeding seasons (birds had no nesting opportunities but bred recently; chicks were removed from aviaries). We focused on pre-breeding and post-breeding seasons for this study, because social interactions were difficult to collect during the breeding season (e.g. due to incubation and parental care occurring within nest-boxes). In each year (2017–2019), we started tracking colonies on the first day when unfamiliar males and females were introduced into the aviaries. We used the same individuals in 2017 and 2018, but as part of a larger experiment [5], we swapped males across aviaries to ensure that they were unfamiliar with members of the other sex at the start of each year's data collection. In 2019, we used the offspring from the 2018 breeding season, again ensuring that all males and all females were unfamiliar with each other. While during the pre-breeding season colonies contained a mix of familiar (within sex) and unfamiliar (between sex) individuals, familiarity was more uniform in post-breeding seasons (as all birds had been together for at least three months). Thus, we analysed these two seasons independently, allowing us to ensure that our findings are robust to colony history (which can also drive differences in behaviours among groups; [31,32]).

### 2.2. Tracking data

All adults were identifiable by a numbered aluminium ring, a unique combination of coloured rings and a backpack containing a unique barcode [29]. In each aviary, 8-megapixel cameras (Module V2, RS Components Ltd and Allied Electronics Inc.) controlled by a Raspberry Pi3 Model B (Raspberry Pi Foundation) were mounted above three areas: the main food source (feeder), one perch commonly used for courting and copulation (copulation perches) and the two perches (social perches) commonly used for resting and social interactions (such as allopreening and sitting in body contact). We used images from these cameras (one image every 3 s), from dawn until dusk, each day throughout the experiment. The identity (barcode number) of the birds present was extracted from each image using the Pinpoint [33] library in Python. The output contained the identity, time, location on the perch and orientation of detected bird, for each camera on each day. Social perches were large enough so that all members of the colony could (and regularly did) fit all at once.

We used data collected in two periods in each of our aviaries (figure 1; electronic supplementary material, table S1): (i) the last 10 days of pre-breeding seasons (approximately 20 days after the birds were introduced, to allow relationships among individuals to establish) and (ii) the entire post-breeding periods (14, 10 and 28 days in 2017, 2018 and 2019, respectively, after chicks were removed; electronic supplementary material, table S1). The number of days in the post-breeding seasons varied across years because of the design of the long-term study [5]. We treated the dataset from pre-breeding seasons and that from post-breeding seasons independently from each other, providing us with a complete replicate of the study (results from pre-breeding seasons are given in the main text; results from post-breeding seasons are given in the main text and electronic supplementary material).

### 2.3. Creating social networks

Previous studies have highlighted the potential importance of distinguishing between interactions and associations in studies of animal social behaviour [34–36]. We therefore used the automated tracking data to generate daily social networks in two social contexts: affiliative interactions (interaction networks) and foraging associations (association networks). Interaction networks were generated from the data from social and copulation perches, with edges representing the tendency for two birds to ‘clump’ given their co-presence on the perch. Clumping is an affiliative interaction comprising two individuals perched in body contact, and was defined (following [5]) as cases where two birds' barcodes were detected less than 80 pixels apart (one body-width). Edge weights were calculated by dividing the number of frames in which two adults were clumped by the number of frames that both birds were detected on the same perch (a strict version of the simple ratio index, see below [30,37]). The strict simple ratio index captures individuals’ decisions about who to clump with among the individuals with whom they are associated, thereby more explicitly extracting their preference in the main context where interactions take place (also see [5]). Under the settings of our aviaries, it is possible for the entire colony to be on the same perch at the same time, thus clumping of sets of individuals on the perch do not exclude other individuals from participating in clumping with either of the two individuals. Thus, interaction networks explicitly capture an individual's social choices in terms of interacting versus not with a given social partner given that both its potential and realized partners are present on the same perch (independently of their tendency to associate). Because body contact is mutual, edges were undirected. Association networks were constructed using data from feeding tables and represented individuals' tendency to move together in the same foraging flock. Edges in association networks were also undirected and were calculated using the simple ratio index [38] (the number of frames in which both focal adults were detected on the same feeding table divided by the number of frames in which at least one of the two focal adults was detected on a given feeding table). Association networks were created in R [39] using the package *asnipe* [40] and interaction networks were created by customizing the *asnipe* get_network function.

### 2.4. External drivers of social network structure

We captured and incorporated information about the following external drivers of social network structure in our data analysis.

#### 2.4.1. Group size

We quantified group size as the number of individuals in the aviary each day using the records from our animal keeping.

#### 2.4.2. Group composition

Birds differed in age across years. All birds in 2017 were 1 year old, and 2 years old in 2018. These then bred, and we used their offspring (1 year old) in 2019. Each colony started with equal sex ratio.

#### 2.4.3. Ecological drivers

We collected three measures to characterize ecological conditions on each day: daily mean temperature, daily mean humidity and daily cloud coverage. We collated these data from the DWD Climate Data Center in Germany [41], taking the data available for the nearest weather station (ID: 02712). As humidity data were not available for 1 day during the study period, we assigned the mean value from the corresponding year.

#### 2.4.4. Physical environments

The block of outdoor aviaries (electronic supplementary material, figure S1) consisted of five aviaries on each of two sides (northeast, southwest). Aviaries in the southwest side faced into a forest and were therefore darker with a lower temperature than those in the northeast side. The latter faced into an opened area with more disturbances. Each year each colony was randomly assigned to a side of the aviary, and it stayed on the same side throughout the year.

The aviary space available to each colony also differed in size between the first and latter 2 years. Each colony was housed in a single aviary (3 × 4 × 3 m) in 2017, but these aviaries were doubled in size by joining adjacent aviaries (6 × 4 × 3 m) in the two subsequent years. Thus, the density of birds per unit of space was halved in the latter 2 years of the study (and the number of perches and feeding tables available to each colony was doubled).

#### 2.4.5. Methodological factors

The same automated camera detection system was used across all aviaries and years. However, weather conditions (e.g. lighter and darker conditions) and variation in the barcode state (e.g. dirty barcodes were less detectable, and barcodes became dirtier over time) can generate differences in the number of detections of each individual and the total number of detections each day. This broadly corresponds to variation in observation effort or detectability that is a pervasive issue in all observational studies.

### 2.5. Social network metrics

We characterized the daily interaction and association network structure of each colony using seven network metrics spanning three social scales (local, intermediate and global scales), producing one value per metric per colony per day. We selected these seven social network metrics to cover the most commonly used metrics in the studies of behavioural ecology [42]. Below we present a brief description of each metric (also see [35,42,43]).

Local-scale connectivity:

(i) Mean weighted degree as the average of the sum of the association or interaction rates (i.e. the edge weights) for each bird in the aviary.
(ii) Mean binary degree as the average of the number of edges connected to an individual (i.e. the number of distinct individuals that a focal individual was observed associating with or interacting with).
(iii) Mean edge weight as the average of the interaction or association rates (the edge weights, including zeros) across all pairs of individuals.

Intermediate-scale metrics capturing how neighbours are interconnected:

(iv) The coefficient of variation of each individuals’ association or interaction rates with other members from its colony (CV edge weight), which is a measure of social differentiation with higher values representing more differentiated relationships (more small and more large values, independent of the mean).
(v) Edge density as the ratio of the number of edges present to the number of possible number of edges in the colony [(*N* × (*N* − 1)/2].

Global-scale measures capturing how each colony is connected overall:

(vi) Mean path length as the average of the shortest number of ‘hops’ needed to connect each individual to everyone else in the colony.
(vii) Diameter as the maximum of the shortest path lengths connecting any two members of the same colony.

We calculated mean weighted degree, mean edge weight and the coefficient of variation directly from the weighted association matrix, and the mean binary degree and edge density from the binarized network (i.e. if any interaction was recorded, then an edge was present). We calculated the mean path length and diameter using the *igraph* package [44], which we applied to the weighted versions of the networks. Since global-scale network measures produce infinite values when there are disconnected components (e.g. two or more individuals were connected to each other but not to the rest of the colony members), we applied the following steps. First, we determined the smallest edge weights for each of interaction and association networks by taking the smallest edge weight values from all individuals whose edge weight was not zero (i.e. detected together at least once) during the study periods. Next, for each daily network, we randomly sampled one individual from each cluster and added the smallest edge weight to connect these two individuals, and determined global-scale network measures (value 1). Then, we compared this value with global-scale network measures with keeping only one cluster with the biggest number of individuals that was detected (value 2). We repeated these steps 100 times and determined the average differences between values 1 and 2, and determined the corrected global-scale measures by adding these average differences to value 2. This corrected global-scale measures were then used for subsequent analyses. All analyses were conducted in *R* [39].

### 2.6. Data analysis

#### 2.6.1. Baseline differences in social network structure between colonies

We first tested whether colonies expressed consistent differences in network metrics without accounting for external drivers. We fitted each daily network metric as a response variable and colony ID as a random effect in linear mixed effect models (herein *uninformed models*) using the *brms* package [45]. We determined colony repeatability, i.e. how much of the variance can be explained by the colony identity, from the outcome of these uninformed models using *performance* R package [46]. To determine confidence intervals of repeatability estimates, we repeated the same processes 1000 times on bootstrapped data. We kept the same model effect structure for each of the seven network metrics. All analyses were conducted on each network type, and pre- and post-breeding networks, separately, using data from all 12 colonies (i.e. data from all three years combined).

#### 2.6.2. Drivers of colony-level differences in social network structure

We next fitted informed models using the same approach as above but controlling for external drivers (group size, group composition, ecological differences, physical environment and methodological differences). Specifically, daily colony size, age of the individuals, daily mean temperature, daily mean humidity, daily mean cloud coverage, aviary aspect, aviary size and daily camera detection for each colony were added as fixed effects (numeric effects were scaled and centred). We fitted date (including year, i.e. YYYY-MM-DD, pre-breeding networks: 30 levels; post-breeding networks: 52 levels) and colony ID (12 levels) as random effects. Again, we fitted the same model structure for each network type, time period and network metric.

The informed models allowed us to test how much variation in each of the measures of colony-level social networks can be explained by our external drivers. We used the brm function [47] in *R* [39] to determine the variance explained by all fixed and random effects and residual variance for each model, and to calculate the marginal and conditional *R*^2^. The marginal $R_{M}^{2}$ captures how much of variance in response variable was explained by fixed effects, and the conditional $R_{C}^{2}$ captures how much of variance was explained by fixed and random effects together. Multi-collinearity in each model was tested using the vif function from the *performance* package [46] in R [39], and we did not detect any problematic collinearity. We also plotted the relationship between each predictor, e.g. ‘camera detection (scaled)’, and each social network metric. For these plots, we calculated corrected values of network metrics (*y*-axis; labelled as *corrected social network metrics*) by subtracting the contribution of all other predictors from the data.

#### 2.6.3. Consistent differences between colonies after controlling for external drivers

We determined whether colony-level social network structure exhibited consistent differences among groups after controlling for external drivers. For this, we extracted the repeatability attributable to colony ID from the informed model using *performance* package [46]. We repeated the procedure on 1000 bootstrapped versions of the data to estimate confidence intervals.

## 3. Results

We collected data over 24 days in 2017 (10 days and 14 days for pre- and post-breeding seasons, respectively), 20 days in 2018 (10 days and 10 days for pre- and post-breeding seasons, respectively) and 38 days in 2019 (10 days and 28 days for pre- and post-breeding seasons, respectively; see electronic supplementary material, table S1). Across the study period, mean temperature ranged from a minimum of 0.0°C to a maximum of 25.6°C, mean humidity ranged between 48.1% and 92.7%, and cloud coverage was distributed from 0 (no cloud) to 8 (fully clouded). The number of detections across all individuals in an aviary on a given day ranged from 6833 to 404 605 for social and copulation perches together, and from 2454 to 71 142 at feeding tables. These data resulted in a total 654 unique networks (i.e. four colonies each year × two network types × 82 days; with two daily foraging networks missing due to camera issues).

### 3.1. Colonies express consistent differences in social network structure when using uninformed models

Repeatability of colony ID, based on the uninformed models fitted with colony ID alone, was moderate to high for most local- and intermediate-scale metrics calculated from pre-breeding interaction networks (*R*: 0.262–0.813), while global-scale metrics had low repeatability (*R*: 0.035 for mean path length and *R*: 0.003 for diameter). Repeatability of colony ID based on the uninformed models was high across most metrics calculated from pre-breeding association networks (*R*: 0.456–0.834, but *R*: 0.268 for diameter). Overall, consistency in colony ID, based on the uninformed models, was higher in pre-breeding association networks than in pre-breeding interaction networks (table 1). For post-breeding networks repeatability values were higher in interaction networks than those in association networks (interaction networks *R*: 0.372–0.897; association networks *R*: 0.197–0.697; electronic supplementary material, table S2), when using uninformed models.

**Table 1.** Repeatability of colony ID in pre-breeding colony-level social network metrics using uninformed models. Repeatability values (*R*) were calculated using uninformed models containing only colony ID (12 levels) as a random effect. These models generate a baseline estimate of how repeatable colonies were when all potential drivers are combined. Post-breeding network results are provided in the electronic supplementary material, table S2.

		interaction network (*n* = 120)			association network (*n* = 118)		
scale	social network metric	*R*	l-95% CI	u-95% CI	*R*	l-95% CI	u-95% CI
local	mean weighted degree	0.813	0.774	0.888	0.762	0.705	0.859
	mean binary degree	0.543	0.471	0.700	0.834	0.793	0.912
	mean edge weight	0.262	0.198	0.516	0.760	0.689	0.859
intermediate	CV edge weight	0.435	0.358	0.644	0.581	0.499	0.761
	edge density	0.549	0.477	0.711	0.456	0.336	0.759
global	mean path length	0.035	0.028	0.354	0.506	0.434	0.706
	diameter	0.003	0.002	0.293	0.268	0.196	0.625

### 3.2. Colony-level differences in network structure are consistently shaped by external factors

The informed model, which controlled for external drivers, explained a high proportion of the variation in pre-breeding colony-level network metrics ($R_{C}^{2}$: 0.639–0.926 in pre-breeding interaction networks; $R_{C}^{2}$: 0.462–0.898 in pre-breeding association networks, table 2). External drivers alone (i.e. the marginal *R*^2^, excluding random effects date and colony ID) typically explained a substantial proportion of the variation in pre-breeding colony-level network metrics (interaction network $R_{M}^{2}$: 0.192–0.705; association networks $R_{M}^{2}$: 0.082–0.626; table 2). We found qualitatively similar results for post-breeding networks (electronic supplementary material, table S3). Although the relative contribution of those predictors was not equal across all metrics and network types, network structure could be relatively well explained by external drivers across all three scales.

**Table 2.** Marginal ($R_{M}^{2})$ and conditional ($R_{C}^{2})\;$variances explained by informed models applied to pre-breeding seasons colony-level interaction and association network metrics. Variance explained for post-breeding networks are presented in the electronic supplementary material, table S3. Detailed outputs of the informed models are given in the electronic supplementary material, tables S4–S7.

scale	metric	interaction network (*n* = 120)		association network (*n* = 118)	
		$R_{M}^{2}$	$R_{C}^{2}$	$R_{M}^{2}$	$R_{C}^{2}$
local	mean weighted degree	0.705	0.856	0.369	0.896
	mean binary degree	0.533	0.924	0.626	0.832
	mean edge weight	0.360	0.670	0.371	0.898
intermediate	CV edge weight	0.386	0.670	0.441	0.898
	edge density	0.507	0.926	0.098	0.594
global	mean path length	0.240	0.666	0.304	0.811
	diameter	0.192	0.639	0.082	0.462

**Figure 2.**
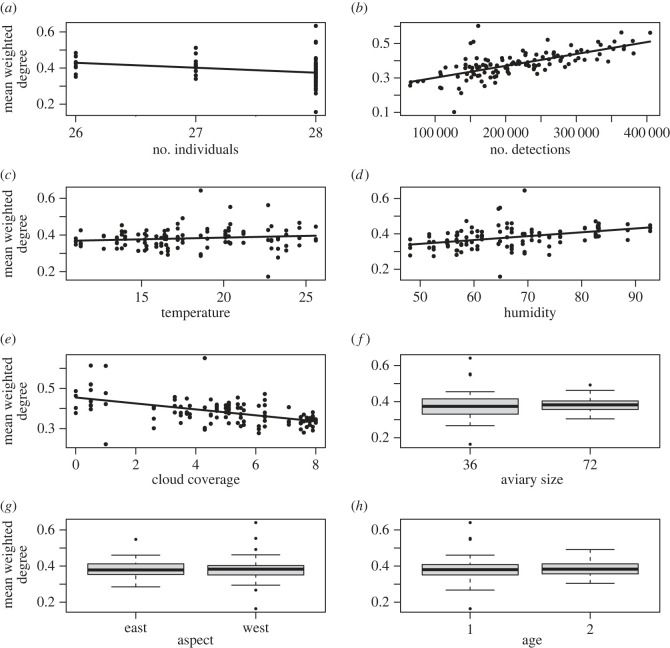
The effect of each predictor on mean weighted degree in pre-breeding interaction networks. Each panel corresponds to a fixed effect in the informed model and shows the relation between that effect and the mean weighted degree after correcting for all the other effects. Black dots (*a*–*e*) represent corrected daily mean weighted degree (i.e. after subtracting the effects of other fixed and random effects from the raw values) for each colony on each day (*n* = 120), and lines (*a*–*e*) show the predicted fit from the informed model. Boxplots (*f*–*h*) show the median, inter-quartile range and outliers for each level when fixed effects were coded as factors (after correcting the raw data for the effects of other fixed and random effects). Results for all metrics are available in the electronic supplementary material, §§2–5.

Covariates affected colony-level network metrics in different directions, and the direction of their effects could differ across network types (electronic supplementary material, tables S4–S7 for all results, and §§2–5 for visualizations). For example, more camera detections corresponded with a higher mean weighted degree in pre- (figure 2) and post- (electronic supplementary material, figure S3.1) breeding interaction networks but a lower mean weighted degree in pre- and post-breeding association networks (electronic supplementary material, figures S4.1 and S5.1). When the daily mean temperature was higher, the mean binary degree in pre-breeding interaction networks was higher (electronic supplementary material, figure S2.1), but the direction of this effect was reversed in the post-breeding interaction networks (electronic supplementary material, figure S3.2), and there was no effect of temperature on pre- and post-breeding association networks (electronic supplementary material, figures S4.2 and S5.2). Thus, how environmental effects impact network structure may vary across studies, within study or across different network types. How strong each covariate drove each colony-level network metric also differed between pre- and post-breeding seasons, though in general the direction of each covariate remained similar within each network type (electronic supplementary material, tables S4–S7).

### 3.3. Colonies exhibit consistent differences in network structure after controlling for external drivers

After controlling for the effects of external drivers, colonies still exhibited consistent differences in social network structure across network types for local- and intermediate-scaled measures in pre-breeding interaction networks (*R* = 0.361–0.562 but 0.135 in mean edge weight, table 3) and post-breeding association networks (*R* = 0.193–0.500), and for all metrics in pre-breeding association networks (*R* = 0.329–0.796) and metrics in pre-breeding association networks (*R* = 0.329–0.796), and for one of local-scaled measures in post-breeding interaction networks (mean weighted degree: *R* = 0.462; full results in table 3; electronic supplementary material, table S9).

**Table 3.** Repeatability of colony ID in pre-breeding colony-level social network metrics after controlling for external drivers. Repeatability values were calculated as per table 1 but with covariates, thereby estimating how repeatable colonies were in terms of their social behaviours alone (table 2 for the $R^{2}$ from these models). Post-breeding results are presented in the electronic supplementary material, table S9. The symbol (*) in the repeatability columns highlights changes in repeatability between the informed and uninformed model that differed by more than 0.1 (relative to uninformed model).

		interaction network (*n* = 120)			association network (*n* = 118)		
scale	metric	*R*	l-95% CI	u-95% CI	*R*	l-95% CI	u-95% CI
local	mean weighted degree	0.391*	0.236	0.674	0.796	0.668	0.874
	mean binary degree	0.540	0.453	0.713	0.537*	0.261	0.797
	mean edge weight	0.135*	0.057	0.428	0.793	0.669	0.879
intermediate	CV edge weight	0.361	0.226	0.696	0.448*	0.294	0.677
	edge density	0.562	0.456	0.742	0.533	0.268	0.795
global	mean path length	0.009	0.006	0.430	0.584	0.390	0.757
	diameter	0.003	0.001	0.284	0.329	0.100	0.633

In pre-breeding interaction networks (table 3), controlling for covariates reduced colony-level repeatability in two local-scale metrics (mean weighted degree and mean edge weight) and one intermediate-scale metric (CV edge weight), while other metrics stayed the same. For example, colony-level repeatability in mean weighted degree decreased from 0.705 (table 1) to 0.391 (table 3), while diameter (global-scale metric) stayed low (from 0.003 to 0.003), after controlling for other effects. In pre-breeding association networks (table 3), one local-scale metric (mean binary degree) and one intermediate-scale metric (CV edge weight) changed in repeatablities. Repeatability of colony ID for five out of seven network metrics remained similar after controlling external drivers. Most of the repeatability estimates in both network types, e.g. mean binary degree in interaction network (*R* = 0.540) and association network (*R* = 0.537), pre-breeding edge density in the interaction network (*R* = 0.562) and pre-breeding mean path length in association network (*R* = 0.533), remained relatively high.

In post-breeding networks (electronic supplementary material, table S9), repeatability for all network metrics decreased for interaction network after controlling for external drivers. However, the repeatability of post-breeding mean weighted degree in both network types (interaction network *R* = 0.462, association network *R* = 0.496), as well as post-breeding CV edge weight (*R* = 0.441) and mean path length (*R* = 0.430) in the association network, remained relatively high.

## 4. Discussion

**Figure 3.**
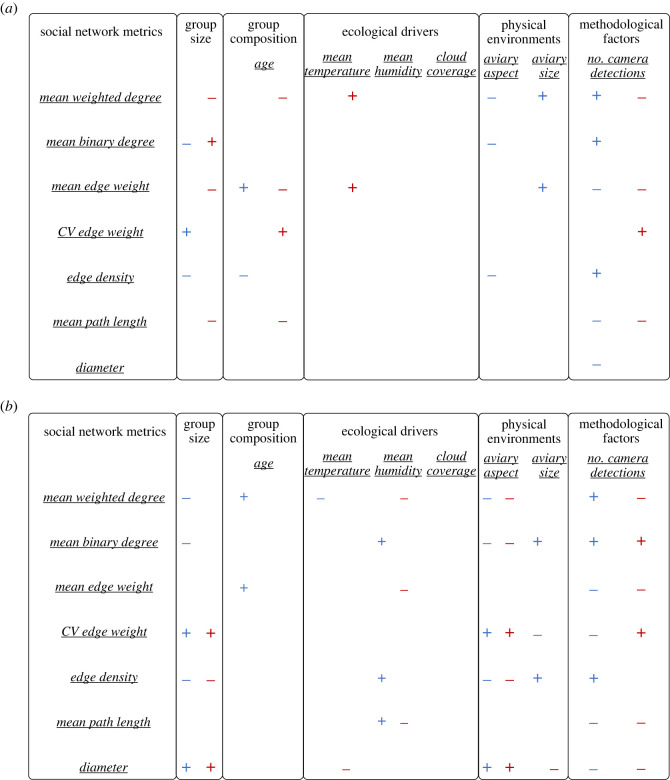
Summary of effects from each driver. Symbols (+/-) show the direction of each external drivers to corresponding colony-level (*a*) interaction and (*b*) association social network metrics. Blue and red represent interaction and association networks, respectively. Only significant estimates whose credible intervals did not overlap with zero are shown in this figure.

Using daily interaction and association networks across 12 colonies (a total of 238 and 416 daily networks in pre-breeding seasons and post-breeding seasons, respectively), we showed that colony-level repeatability estimates of most commonly used group-level network metrics are (i) consistently different, (ii) affected by a range of external drivers, and (iii) can remain highly repeatable within colony even after accounting for external drivers. We further found that external drivers can vary in their contributions to group-level network metrics, affecting different network types (interaction versus association networks) in different directions (figure 3), but usually shape network metrics consistently across different seasonal contexts (pre- versus post-breeding seasons). Together, these results confirm that animal social groups can exhibit consistent variation in social network structure, that network structure is shaped by a range of different drivers, and that groups still express consistent differences once these drivers are accounted for.

Our study demonstrates that there can be consistent group-specific differences in network structures even after controlling for external drivers. In doing so, we can reject the null hypothesis that ‘identical groups do not vary in social structures when placed under identical environments'. However, since it is natural to have some environmental or social differences among groups in both wild and captive populations, it should also be generally expected that any observed differences in group-level network metrics are substantially inflated when using models that do not control for a full range of external drivers. However, given sufficient data, we have demonstrated that models controlling for external drivers can still recover significant repeatability estimates, even when the drivers have strong effects on network structure.

We found that local-scale metrics were typically more likely to be repeatable than global-scale metrics, especially for interaction networks. The lower repeatability among global-scale metrics highlights that even when groups behave in consistent ways, the emergent global properties of networks are highly dynamic, which is because global metrics can often be affected by changes in just one or two edges [7]. The higher repeatability in global-scale metrics for associations is unlikely to be because interactions are inherently more variable within groups. Rather, it is likely to be a methodological artefact, arising from the ability to observe individuals with more associates than with interaction partners, making the latter sparser and more sensitive to the presence or absence of a few edges. This effect is likely to play a role in all studies using interaction networks.

Our study also highlights that animal groups can develop distinct network structures in relatively short time frames. Our pre-breeding networks were recorded on average 20 days after birds were introduced into aviaries, whereas post-breeding networks were from 98 to 152 days after introduction (electronic supplementary material, table S1). Yet, network metrics from pre-breeding seasons were on average equally repeatable to post-breeding networks. Recent studies on schools of fish also found a rapid divergence of group-level properties over just a few days under laboratory conditions [48]. Thus, our study adds to a growing body of evidence that differences are emergent to the groups, including groups from relatively anonymous societies (like schooling fish) and those that have strong and consistent social bonds (such as socially monogamous zebra finches).

Our results further show that it is difficult to predict whether (or how) any one external driver would affect a given metric in a given network type (association networks versus interaction networks, i.e. biological contexts; figure 3). For example, group size had significant but different effects depending on network types and seasonal context (figure 3; electronic supplementary material, tables S2–S5). Individuals in larger groups appeared to be choosier in their interaction partners, but less choosy about who they foraged with. This is in line with previous theoretical and empirical studies [49,50], suggesting that group sizes can account for individuals' behavioural patterns (e.g. [51–53]) and also variations in group-level behavioural patterns and outcomes [49,54]. More studies are needed to determine whether there is any consistency in the strength or direction of external drivers on group-level network properties, as this knowledge would make important contributions to our understanding of how changes in the environment may impact the social structure, and behaviour, of the animals living within them.

Group composition (age) also had significant effects on multiple colony-level network metrics. Colonies consisting of older individuals were typically more exclusive (stronger social interaction with smaller number of individuals). These results are in line with previous studies in other species (e.g. [55]), despite the range of differences in ages (2 versus 1 year) being relatively small (20% or less of the estimated lifespan of captive zebra finches). Our results also extend previous studies that found that personality [2] and sex [1,3,18] can explain how individuals behave, and that these can lead to group-level differences in network structure. Given the range of factors that can contribute to group composition (e.g. experience, size), it might be challenging to always eliminate the contribution that composition will make to the structure of any animal social network.

Ecological conditions (daily temperature, daily humidity and daily cloud coverage) also influenced network metrics. In response to weather, individuals modified several aspects of who they associated and interact with, and how frequently, thereby underpinning day-to-day differences in the colony-level social behaviour estimated from the data. These results confirm the general expectation that individuals modify their behaviour as ecological conditions change [14,15,20,21,56,57]. Colony-level network metrics were also influenced by more stable physical features of environments. Changes in the available space consistently altered the structure of interaction networks (although these changes were not necessarily carried over into foraging). These findings are consistent with a recent study finding that red-backed fairywrens (*Malurus melanocephalus*) exhibited higher network connectivity (albeit in an association context) when the available habitat decreased due to recent fires [28]. Thus, we expect that ecological conditions and physical space will affect almost all animal social networks in some way.

Methodological differences also had strong effects across multiple network metrics. These results were somewhat surprising to us and worrying for researchers. Our automated tracking systems consistently collected more than 10 000 detections per aviary per day, corresponding to well over 300 detections per individual (on average). This sampling effort is substantially higher than what the literature recommends is needed to construct robust social networks (at least 20 observations per dyad [58]), yet the number of camera detections still had a remarkably clear effect across the range of network metrics. One caveat to this result might be that camera detections indirectly correlated with some environmental factors in ways that are difficult to disentangle. For example, if camera detections are a clearer signal of ecological conditions than those we had available from the weather station, then it could be that our camera detections also include some of the variance attributable to weather. However, we did not find strong collinearity among our variables. This subject warrants further verification using groups in more stable conditions (e.g. indoors) and/or using subsampling approaches to standardize the number of detections.

Overall, our study shows that several sources of variation (group size, group composition, ecological differences, physical environments and methodological differences) can substantially impact the inferred and expressed structure of animal social network. Ignoring the role that external factors will have could therefore easily lead to spurious (over-estimated) between-group differences in social network structures [11]. On a more positive note, we found that our estimates of between-group differences in social network structure (repeatability) were not universally decreased when controlling for external factors (including ‘date’, which should have accounted for other latent environmental variables), suggesting that repeatability estimates are likely to be quite robust to their presence. However, this is likely to be substantially facilitated by having collected data under replicated conditions (i.e. four colonies per day), which is rarely feasible (especially as networks are generally averaged over many days' worth of data). Further, it is important to note that while factors might act as nuisance effects in some cases, for example, if the aim is to study consistent group-level behaviour that might be attributable to cultural differences [6,11], in others they might represent important predictors of meaningful variation in network structure, for example in the study of disease dynamics [59]. We therefore suggest that researchers carefully consider the different sources of variation that could impact their observed networks, and whether these represent confounding or informative effects. Further, we hope that more studies focus on characterizing the drivers of between-group differences in social network metrics, including evaluating whether the remaining variance attributable to colonies represents a true ‘group-level effect’.

## Data Availability

All data and code to replicate our analysis are available at https://doi.org/10.6084/m9.figshare.c.6742204 [60].
